# Internal focalization influences real-life empathy: a literary contribution to wellbeing

**DOI:** 10.3389/fpsyg.2026.1968457

**Published:** 2026-10-01

**Authors:** Angus Fletcher, Mark Matthews

**Affiliations:** The Ohio State University, Columbus, OH, United States

**Keywords:** empathy, focalization, forgiveness, literature, wellbeing

## Abstract

Literature has often been hypothesized to cultivate an empathy that persists after reading, yielding real-life wellbeing benefits. But the subtle complexity of literature’s aesthetic form has made this hypothesis challenging to test. To simplify the challenge, we isolated a single formal technique—internal focalization—historically used by literary fictions that focus on a specific kind of empathy: forgiveness. We incorporated internal focalization into a fictional narrative, and across three preregistered studies (*N* = 371; *N* = 449; *N* = 743), we measured the effect on participants’ likeliness to forgive. In Study 1, we found that internal focalization prompted increased likeliness to forgive fictional transgressors. In Studies 2 and 3, we replicated this finding and further demonstrated a link between forgiveness of fictional transgressors and forgiveness of real-life transgressors. These findings suggest that literature’s wellbeing benefits can be assessed by parsing its form into separate techniques.

## Introduction

Literary fiction has long been credited as a source of various kinds of empathy, from the compassion (*ἔλεος*) that Aristotle located in dramatic tragedies to the “fellow feeling” that Adam Smith ascribed to novels. Meanwhile, non-literary fiction has been blamed by authors such as Jane Austen and George Bernard Shaw for creating false empathy, an egocentric sentiment that leads to personal isolation, emotional imbalance, and social harm. Since empathy plays a role in personal and communal wellbeing, this distinction between literary and nonliterary variants has seemed a fruitful starting-point for exploring the mental health benefits of literature (Mar et al., 2006; Mar et al., 2009; Bal and Veltkamp, 2013; Kidd and Castano, 2013; Oatley, 2016; Denham, 2024). However, the distinction has proved challenging to test empirically, due to literature’s formal subtlety and complexity (Eekhof et al., 2022). So nuanced and intricate are the methods of literary writing that they can seem ineffable or entirely subjective: the novels celebrated by Smith are regarded warily by Austen, while Shaw condemns many classic dramas as nonliterary “trash of the lowest melodramatic order.” As a result, experimental attempts to link literature to empathy have proved inconclusive (e.g., Panero et al., 2017; Dodell-Feder and Tamir, 2018; Samur et al., 2018; Kidd and Castano, 2019; Takahashi et al., 2023; Wimmer et al., 2024).

In search of a fresh approach, we set aside the prior experimental method of using modern editors (such as the judges of the National Book Awards) to define what constitutes literature. Instead, in keeping with recent research into the origins of literature (Fletcher, 2021), we characterized literary form as a biological—specifically, anthropological—phenomenon. That is, we did not treat literary form as a unified (even transcendental) quality. Rather, we treated it as a library of environmentally-situated aesthetic techniques invented in response to a range of distinct human concerns. Some of these concerns were social, historical, or economic: political rights, group status, personal wealth. Others were medical: grief, anger, loneliness, and trauma.

We hypothesized that literary techniques devised in response to medical concerns could translate beyond their point of origin, yielding health benefits in other times and places. One such technique is internal focalization. Internal focalization is part of a subset of literary techniques that focus attention within a narrative (Scholes et al., 2023). It emphasizes the inner feelings, motives, and thoughts of another person or persons, as for example when George Eliot’s 1860 novel *The Mill on the Floss* relates: “The song suggested distinct memories and thoughts, and brought quiet regret in the place of excitement” (For further historical examples, see Fletcher, 2021, Chapter 3). Internal focalization can be found across Greek tragedies, eighteenth-century novels, and Austen and Shaw’s own fictions. In these literary works, internal focalization is frequently positioned as a tool for producing a specific empathy effect: forgiveness. Since forgiveness has been linked, outside of literature, to wellbeing benefits, we concluded that internal focalization was a candidate for testing our anthropological approach to the question of literary form’s contribution to mental health. Rather than attempting to answer the question all at once, it afforded a case study in the effectiveness of parsing the question into narrower ones, in this case: Can internal focalization influence forgiveness?

### Forgiveness

Forgiveness is a process of restoring safety and connection following a transgression (Takaku, 2001). That restoration can be decisional or emotional. Decisional forgiveness is a cognitive-behavioral intention to forgive a transgressor, whereas emotional forgiveness is a reduction in negative emotions towards a transgressor (Worthington et al., 2007; Lichtenfeld et al., 2019; Li et al., 2024). Decisional forgiveness promotes changes in how a person will behave towards a transgressor: If a person decides to forgive, they will be more willing to cooperate with the transgressor in the future. Emotional forgiveness leads to renewed feelings of closeness as well as a greater tendency to forget the transgression experience (Lichtenfeld et al., 2015).

Decisional and emotional forgiveness are more likely when victims trust the remorse of the transgressor (Fincham, 2000; McCullough, 2001; Russell et al., 2025). That trust carries health benefits but also dangers. People who report higher forgiveness have greater levels of personal wellbeing and life satisfaction (Gao et al., 2022), as well as higher quality relationships (Allemand et al., 2007). However, they also run the risk of being harmed again. Victims can become trapped in cycles of harm by a willingness to forgive indiscriminately (Gordon et al., 2004; McNulty, 2011; Golden et al., 2025). One potential value of literature could thus be to facilitate the development of empathy by providing the opportunity to practice forgiveness in a safe, unpressured environment. And one potential value of internal focalization could be to enrich that practice with access to transgressors’ inner thoughts and sentiments, cultivating healthy forgiveness.

### Overview of studies

The present research reports on three studies probing the link between (a) the literary technique of internal focalization and (b) forgiveness for transgressors. Study 1 examined whether the formal use of internal focalization can facilitate forgiveness for literary transgressors. Studies 2 and 3 examined whether facilitating forgiveness for literary transgressors can facilitate forgiveness for real-life transgressors. (By “real-life” we intend the nonfictional world, as it is perceived). We predicted that internal focalization would facilitate literary forgiveness and that literary forgiveness would facilitate forgiveness of real-life transgressors. All three studies received prior ethics approval and were preregistered on OSF.

## Study 1

The goal of Study 1 was to examine whether forgiveness of a fictional transgressor could be facilitated by internal focalization. Because forgiveness often relies on the ability to either (a) attribute the transgression to an external cause (McCullough, 2001) or (b) trust that the transgressor will not commit the transgression again (Fincham, 2000), we also manipulated the transgression scenario to vary internal/external attribution. This yielded a 2 (Attribution: internal, external) x 2 (Focalization: no focalization, internal focalization) between-subjects design. We hypothesized that participants would be more forgiving in the external attribution condition. We further hypothesized that participants would be more forgiving when granted internal focalization. Finally, we expected that the effect of internal focalization would be more prominent in the internal attribution condition, because in that condition, the thoughts and sentiments of the transgressor are more relevant to forgiveness.

### Method

#### Participants

Amazon Mechanical Turk Participants (*N* = 483) participated in exchange for $1.00. Participants were excluded if they did not complete the full survey (*n* = 83) or if they failed basic comprehension check questions (*n* = 29), yielding a final sample of *N* = 371. We did not collect demographic information in this study (or the subsequent two); however, it is expected that our samples reflected the population of Amazon Mechanical Turk Participants at the time: predominantly white, primarily 20–40, slightly more female and of lower socio-economic status (Hauser et al., 2023). Informed consent was completed online at the start of the survey, and participants were assigned to one of four experimental conditions as part of a 2 (Attribution: internal vs. external) x 2 (Focalization: internal focalization vs. no internal focalization) between-subjects design. A power analysis using G*Power suggests a sample of 279 to detect a medium-sized effect (*f* = 0.25) at an alpha of 0.05 for a 2 × 2 Between-Subjects ANOVA at 95% power (Faul et al., 2007; Kang, 2021).

#### Procedure

All participants began by reading a four-part story about *Mary*, an 85-year-old woman who was transgressed by a repairman. (Full story available in stimulus materials file). The story varied depending on the experimental condition to which participants were assigned. In all conditions, participants read the same first two parts of the story.

##### Story Parts 1 & 2: the transgression

In Part 1 of the story, participants read about Mary’s childhood experience of watching her puppy tragically killed by a car. Due to her grief, Mary swore to never own a dog again, but at the age of 85, she decided to heal by adopting a new puppy. After Part 1 of the story, participants reported on the extent to which they felt Mary was *likeable, warm, and compassionate* on a 1 (*Not Very*) – 9 (*Extremely*) scale. These items were highly correlated and were averaged into a composite measure of liking Mary (*a* = 0.94).

In Part 2 of the story, participants read about a day when Mary called a repairman to fix her oven. Mary carefully explains to the repairman the importance of making sure the door is kept shut to keep her puppy inside. Nevertheless, the repairman carelessly leaves the door open and the puppy escapes, causing Mary to collapse in horror.

##### Story Part 3: attribution manipulation

In Part 3 of the story, participants were offered information about the repairman that varied internal/external attributions. In the internal attribution condition, it was implied that the repairman disregarded Mary’s requests to pay attention to the door, suggesting that he was at cause for the transgression. In the external attribution condition, it was implied that the repairman cared about checking the door; however, due to personal issues at home, he became distracted and mistakenly left the door open.

At the conclusion of Part 3, participants completed two items assessing the extent to which the repairman was responsible for the puppy escaping on 1 (*not at all responsible*) – 10 (*entirely responsible*) scale that were averaged together to form a *responsibility* composite (*r* = 0.82, *a* = 0.90). Participants also completed two items assessing the extent to which they felt the door being left open and the puppy getting out were caused by factors outside the repairman’s control on a 1 (*not at all caused by external factors*) – 10 (*entirely caused by external factors*) scale that were averaged together into a *controllability* composite (*r* = 0.77, *a* = 0.87).

##### Story Part 4: internal focalization manipulation

In Part 4 of the story, the repairman apologized to Mary, and the presence of internal focalization was manipulated. In the literary condition, the story was enriched with the literary technique of internal focalization: “The repairman thought to himself, I wish I didn’t leave the door open.” In the nonliterary condition, participants witnessed the repairman apologizing to Mary, but without the literary technique of internal focalization: “The repairman said to Mary, I wish I didn’t leave the door open.” Participants then reported the extent to which they felt they could see into the repairman’s mind on a 1 (*not at all: I do not feel like I know what he is actually thinking*) – 10 (*entirely: I feel like I know exactly what the repairman is thinking*) scale. They also reported on the extent to which they felt certain of what he was thinking on a 1 (*not at all: I have no clue about the repairman’s motives*) – 10 (*entirely: I am certain of his motives for apologizing*) scale. These two items formed an *internal focalization* composite (*r* = 0.71, *a* = 0.83).

##### Forgiveness

Participants reported on both decisional and emotional measures of forgiveness. *Decisional forgiveness* was assessed by (a) asking whether Mary should forgive the repairman, and (b) asking the extent to which participants themselves would forgive the repairman on a 1 (*absolutely not*) – 10 (*absolutely*) scale. These two items were averaged together to form the decisional forgiveness measure (*r* = 0.92, *a* = 0.95). *Emotional forgiveness* was assessed using the affective subscale of the Multi-Dimensional Forgiveness Inventory (Hillman and MacDonald, 2025). Participants were asked to imagine that they were Mary and had decided to forgive the repairman. They then reported how they would feel on nine items. Sample items include, *If I were Mary, thinking about the repairman leaving the door open would make me sad* and *If I were Mary, the repairman would have a lot to make-up for.* These items were answered using a 1 (*strongly disagree*) – 7 (*strongly agree*) scale. The nine items were averaged together to form the emotional forgiveness measure (*a* = 0.92).

#### Transparency and openness

This study’s design, hypotheses, and analysis were preregistered prior to data collection.^1^ We reported how we determined our sample size, all data exclusions, all manipulations, and all measures in this study. The Institutional Review Board of The Ohio State University granted its approval for the experiment prior to the commencement of data collection and the initiation of the experimental procedures.

### Results and discussion

#### Manipulation checks

##### Mary liking

On average, participants reported positive attitudes towards Mary (*M =* 8.03, *SD* = 1.15), which was significantly higher than the scale midpoint of 5, *t(370)* = 50.49, *p* < 0.001, Cohen’s *d* = 2.62, mean difference = 3.03, 95% CI [2.91, 3.14].

##### Transgressor responsibility

A one-way ANOVA showed that participants in the external attribution condition (*M* = 8.61, *SD* = 1.71) did not hold the transgressor any less responsible for the transgression compared to participants in the internal attribution condition (*M* = 8.70, *SD* = 1.68), *F*(1,347) = 0.27, *p* = 0.601, η_p_^2^ = 0.001, 95% CI [−0.45, 0.26].

##### Transgressor controllability

A one-way ANOVA showed that participants in the external attribution condition reported that the transgression was more likely to be caused by factors outside of the transgressor’s control (*M* = 3.65, *SD* = 2.35) compared to participants in the internal attribution condition (*M* = 3.08, *SD* = 2.06), *F*(1,346) = 5.78, *p* = 0.017, η_p_^2^ = 0.016, 95% CI [0.10, 1.04], suggesting that attribution manipulation condition successfully manipulated controllability dimension of attribution.

##### Internal focalization

A one-way ANOVA showed that participants in the literary condition reported greater feelings of access to the repairman’s mind (*M* = 7.22, *SD* = 2.14) compared to participants in the nonliterary condition (*M* = 6.67, *SD* = 2.41), *F*(1,346) = 5.02, *p* = 0.026, η_p_^2^ = 0.014, 95% CI [0.07, 1.03], suggesting that focalization manipulation successfully impacted internal focalization. In sum, the stories created sufficient liking for the victim while also successfully manipulating attribution and internal focalization.

#### Forgiveness

Two separate factorial ANOVAs were used to examine the extent to which the attribution and literary conditions affected decisional and emotional forgiveness.

##### Decisional forgiveness

There was no main effect of internal focalization on decisional forgiveness, *F*(1,365) = 1.13, *p* = 0.288, η_p_^2^ = 0.003, 95% CI [−0.24, 0.79]. There was, however, a main effect of attribution: Participants were more forgiving in the external condition (*M* = 8.23, *SD* = 2.52) compared to the internal attribution condition (*M* = 6.81, *SD* = 2.51), *F*(1,365) = 29.80, *p* < 0.001, η_p_^2^ *= 0.*075, 95% CI [0.91, 1.94]. This main effect was qualified by a significant focalization x attribution interaction, *F*(1,365) = 8.71, *p* = 0.003, η_p_^2^ = 0.023, 95% CI [0.52, 2.58]. In the external attribution condition, there were no differences in forgiveness in the literary condition (*M* = 7.99, *SD* = 2.68) compared to the nonliterary condition (*M* = 8.48, *SD* = 2.24), *F*(1,365) *=* 1.85, *p = 0*.175, η_p_^2^ = 0.005, 95% CI [−1.21, 0.22,]. However, in the internal attribution condition, participants were more forgiving in the literary condition (*M* = 7.33, *SD* = 2.38) compared to the nonliterary condition (*M* = 6.28, *SD* = 2.75), *F*(1,365) = 7.78, *p* = 0.006, η_p_^2^ = 0.021, 95% CI [0.31, 1.79] (see Figure 1).

**Figure 1:**
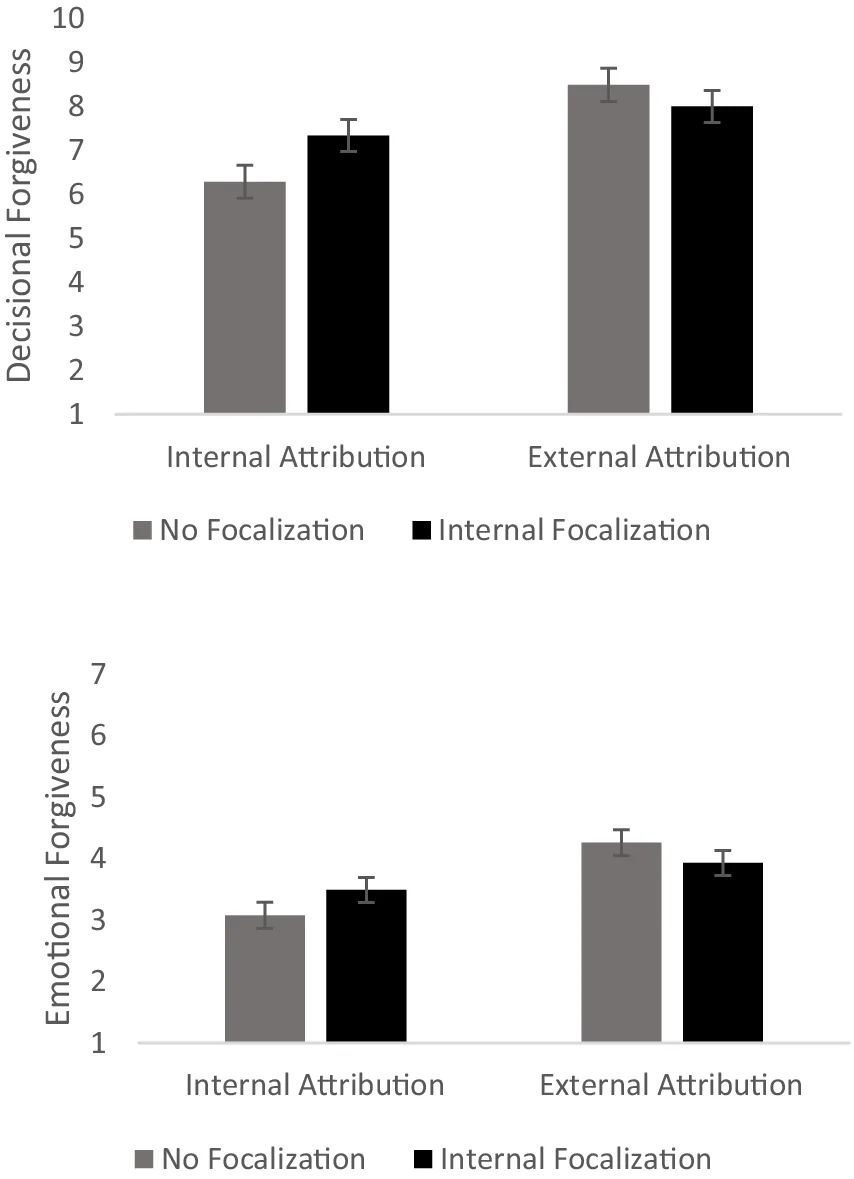
Two-way ANOVAS examining attribution x internal focalization interaction on decisional and emotional forgiveness (Study 1).

##### Emotional forgiveness

Paralleling the findings on decisional forgiveness, there was no main effect of internal focalization, *F*(1,340) = 0.10, *p* = 0.759, η_p_^2^ = 0.000, 95% CI [−0.33, 0.24]. Again, there was a main effect of attribution: Participants were more forgiving in the external attribution condition (*M* = 4.09, *SD* = 1.36) compared to the internal attribution condition (*M* = 3.28, *SD* = 1.36), *F*(1,340) = 30.19, *p* < 0.001, η_p_^2^ *= 0.*082, 95% CI [0.52, 1.10]. This main effect was qualified by a significant focalization x attribution interaction, *F*(1,340) = 6.43, *p* = 0.012, η_p_^2^ = 0.019, 95% CI [0.17, 1.32]. In the external attribution condition, there were no differences in forgiveness in the literary condition (*M* = 3.93, *SD* = 1.34) compared to the nonliterary condition (*M* = 4.26, *SD* = 1.55), *F*(1,340) *=* 2.59, *p = 0*.109, η_p_^2^ = 0.008, 95% CI [−0.73, 0.07,]. However, in the internal attribution condition, participants were more forgiving in the literary condition (*M* = 3.49, *SD* = 1.32) compared to the nonliterary condition (*M* = 3.08, *SD* = 1.20), *F*(1,340) = 3.89, *p* = 0.049, η_p_^2^ = 0.011, 95% CI [0.00, 0.84] (see Figure 1).

As expected, participants were more forgiving in the external attribution condition. Our findings extend previous research on attribution and forgiveness (Takaku, 2001) by demonstrating that external attribution promotes both decisional and emotional forgiveness. In the internal attribution condition, internal focalization facilitated both decisional and emotional forgiveness. These findings suggest that internal focalization facilitates forgiveness following an apology when the transgressor is considered to have been in control of their actions.

## Study 2

The purpose of Study 2 was to replicate the findings of Study 1 and to examine whether greater forgiveness in the literary scenario yielded forgiveness when reflecting on a real-life transgressor. The same literary transgression scenario was used. Because the effects of internal focalization emerged only in the internal attribution condition, only the internal attribution versions of the story were used, yielding a 2 (Focalization: internal focalization vs. no focalization) between-subjects design. A personal past transgression reflection paradigm was added to this study following the literary paradigm. The preregistration for Study 2 can be accessed at https://osf.io/tsaqb/?view_only=4f13eac0fdc740cf9942aa7b9a3627f1.

### Method

#### Participants

Amazon Mechanical Turk Participants (*N* = 505) were recruited for a study involving reading a story and answering related questions for $1.25. Participants were excluded if they did not complete the full survey, including primary dependent measures, or if they failed basic comprehension check questions related to information presented in the story (*n* = 56), yielding a total sample of *N* = 449 for analysis. Informed consent was completed online at the start of the survey. Because of the inherent uncertainties of power analyses for studies with complex effects (e.g., Pek et al., 2024), a large number of participants was sampled.

#### Procedure

All participants began the study by reading the same four-part story about Mary, varied by experimental condition. All participants read the version of the story designed to produce greater internal attributions. Participants were randomly assigned to the literary condition or the nonliterary condition, and they completed the same dependent measures as in Study 1 for the story. In Study 2, participants also completed a personal transgression paradigm after the story.

##### Personal transgression paradigm

Participants completed a personal transgression reflection (adapted from Hillman and MacDonald, 2025), where they reflected on a time when someone close had severely transgressed them. Participants listed the initials of this person, reported the type of relationship and level of closeness, and described the transgression in detail. Next, participants were prompted to answer questions about whether the person had apologized and what would need to happen for them to forgive the transgressor. Participants then were asked to report on one item measuring decisional forgiveness, *Do you feel you have fully forgiven this person,* on a 1 (*absolutely not*) – 10 (*absolutely*) scale. Finally, participants were asked to complete the emotional measure of forgiveness, the 9-item MDI-affective subscale (Hillman and MacDonald, 2025).

#### Transparency and openness

This study’s design, hypotheses, and analyses were preregistered prior to data collection.^2^ The Institutional Review Board of The Ohio State University granted its approval for the experiment prior to the commencement of data collection and the initiation of the experimental procedures.

### Results and discussion

#### Manipulation checks

##### Mary liking

On average, participants reported positive attitudes towards Mary (*M =* 7.96, *SD* = 1.13), *t*(447) = 55.50, *Cohen’s D* = 2.62, 95% CI [2.86, 3.07].

##### Internal focalization

*A* one-way ANOVA showed that participants in the literary condition reported greater feelings of access to the repairman’s mind (*M* = 6.84, SD = 2.21) compared to participants in the nonliterary condition (*M* = 6.17, *SD* = 2.30), *F*(1,419) = 9.35, *p* = 0.002, η_p_^2^ = 0.022, 95% CI [0.24, 1.11]. In sum, the stories provided created sufficient liking for the victim while also successfully manipulating internal focalization.

#### Literary forgiveness (replicating study 1)

For each of the primary dependent measures, a separate One-Way ANOVA was used to examine the extent to which the literary condition affected both decisional and emotional forgiveness.

##### Decisional forgiveness

There was an effect of internal focalization on decisional forgiveness, replicating Study 1, *F*(1,446) = 11.07, *p* = 0.001, η_p_^2^ = 0.024, 95% CI [0.36, 1.39]. Participants were more forgiving in the literary condition (*M* = 6.97, *SD* = 2.59) compared to the nonliterary condition (*M* = 6.09, *SD* = 2.95, see Figure 2).

**Figure 2:**
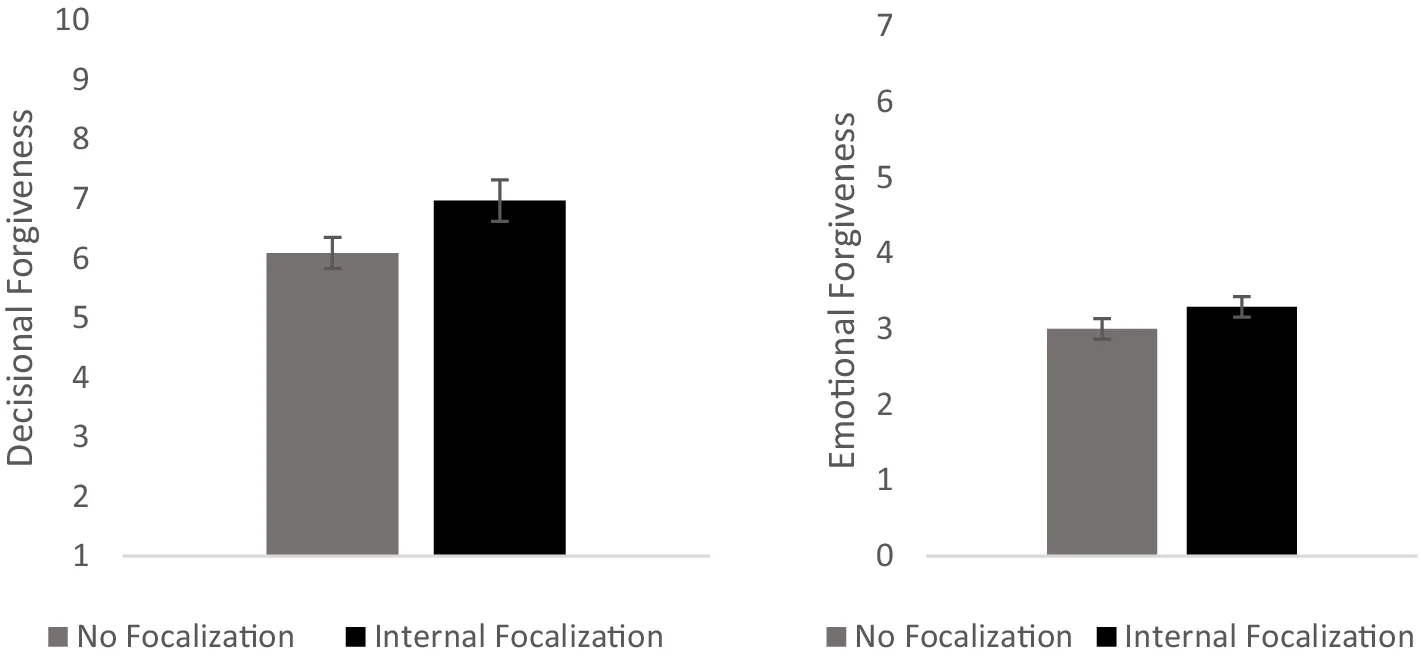
One-way ANOVAS examining the effects of internal focalization on decisional and emotional forgiveness (Study 2).

##### Emotional forgiveness

There was also an effect of internal focalization on emotional forgiveness, replicating Study 1, *F*(1,415) = 4.25, *p* = 0.040, η_p_^2^ = 0.010, 95% CI [0.01, 0.55]. Participants were more forgiving in the literary condition (*M* = 3.29, *SD* = 1.34) compared to the nonliterary condition (*M* = 3.00, *SD* = 1.44, see Figure 2).

#### Real-life transgression forgiveness

It was expected that literary forgiveness would promote real-life forgiveness. To test this hypothesis, two mediation models (Process V4.1, Model 4, Hayes, 2017) were computed to examine the indirect effect of internal focalization on real-life decisional and emotional forgiveness. Internal focalization was entered as the focal predictor (x). Both decisional (M_1_) and emotional (M_2_) literary forgiveness were entered as parallel mediators in the same model. The effects of internal focalization on the literary measures (A paths) correspond to the previously reported analyses, so we only report the effects of the literary forgiveness measures on real-life forgiveness measures (B paths) and the direct and indirect effects of internal focalization on the real-life forgiveness measures (c’ path and indirect AB paths).

##### Real-life decisional forgiveness

Literary decisional forgiveness positively predicted real-life decisional forgiveness, *B* = 0.35, *SE* = 0.07, *t*(376) = 4.97, *p* < 0.001, 95% CI [0.21, 0.49], whereas literary emotional forgiveness did not predict real-life decisional forgiveness, *B* = 0.14, *SE* = 0.14, *t*(376) = 1.00, *p* = 0.319, 95% CI [−0.14, 0.42]. As predicted, internal focalization had a positive indirect effect on real-life decisional forgiveness via literary decisional forgiveness, *B* = 0.16, *SE* = 0.06, *t*(376) = 2.71, *p* = 0.007, 95% CI [0.06, 0.29] (see Figure 3).

**Figure 3:**
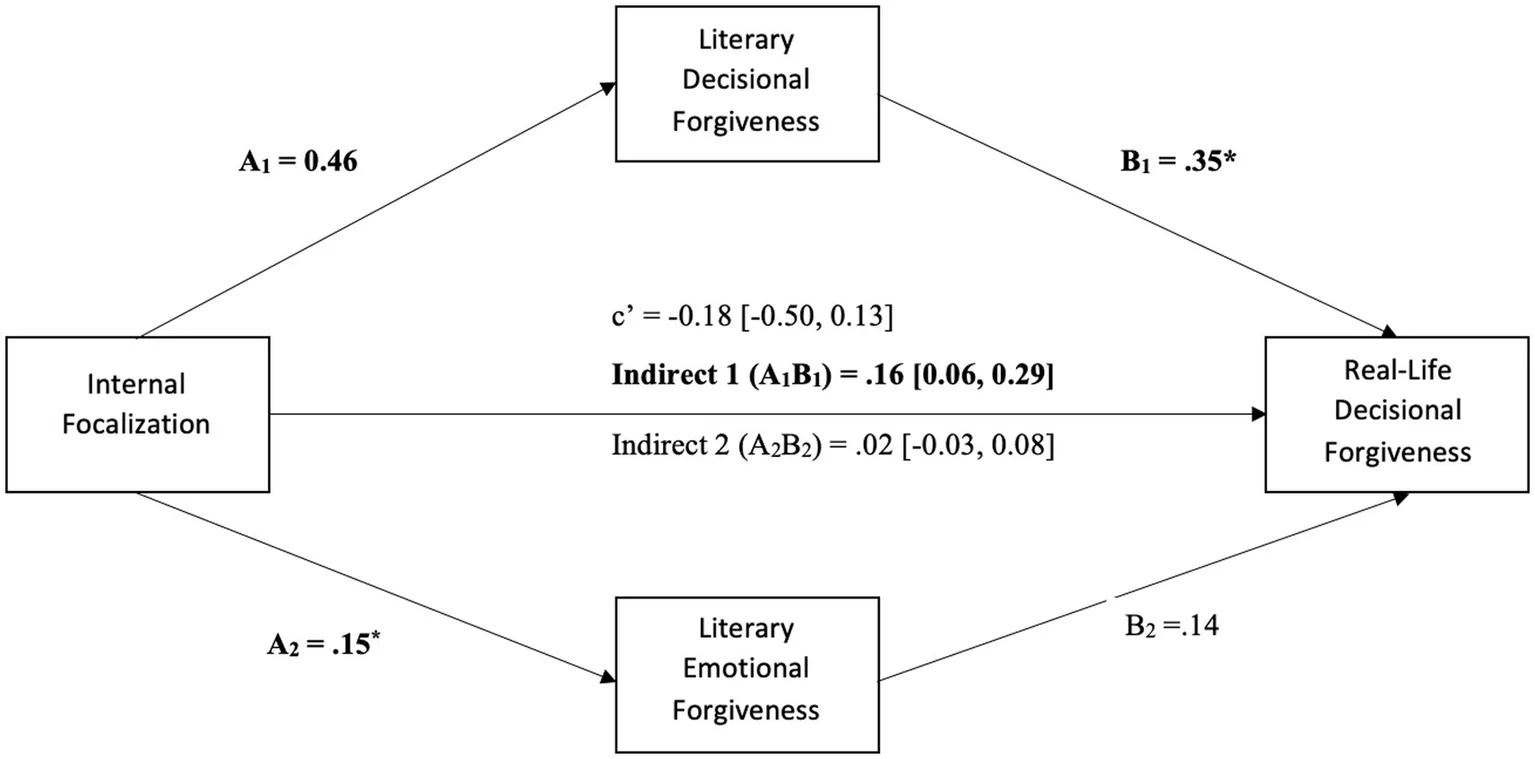
Study 2 mediation model predicting decisional forgiveness (*n* = 380). *p < 0.05.

##### Real-life emotional forgiveness

Literary emotional forgiveness positively predicted real-life emotional forgiveness, *B* = 0.33, *SE* = 0.06, *t*(353) = 5.79, *p* < 0.001, 95% CI [0.22, 0.44]. Literary decisional forgiveness did not predict real-life emotional forgiveness, *B* = 0.04, *SE* = 0.03, *t*(353) = 1.28, *p* = 0.203, 95% CI [−0.02, 0.09]. As predicted, internal focalization had a positive indirect effect on real-life emotional forgiveness via literary emotional forgiveness, *B* = 0.05, *SE* = 0.03, *t*(353) = 1.89, *p* = 0.058, 95% CI [0.00, 0.11] (see Figure 4).

**Figure 4:**
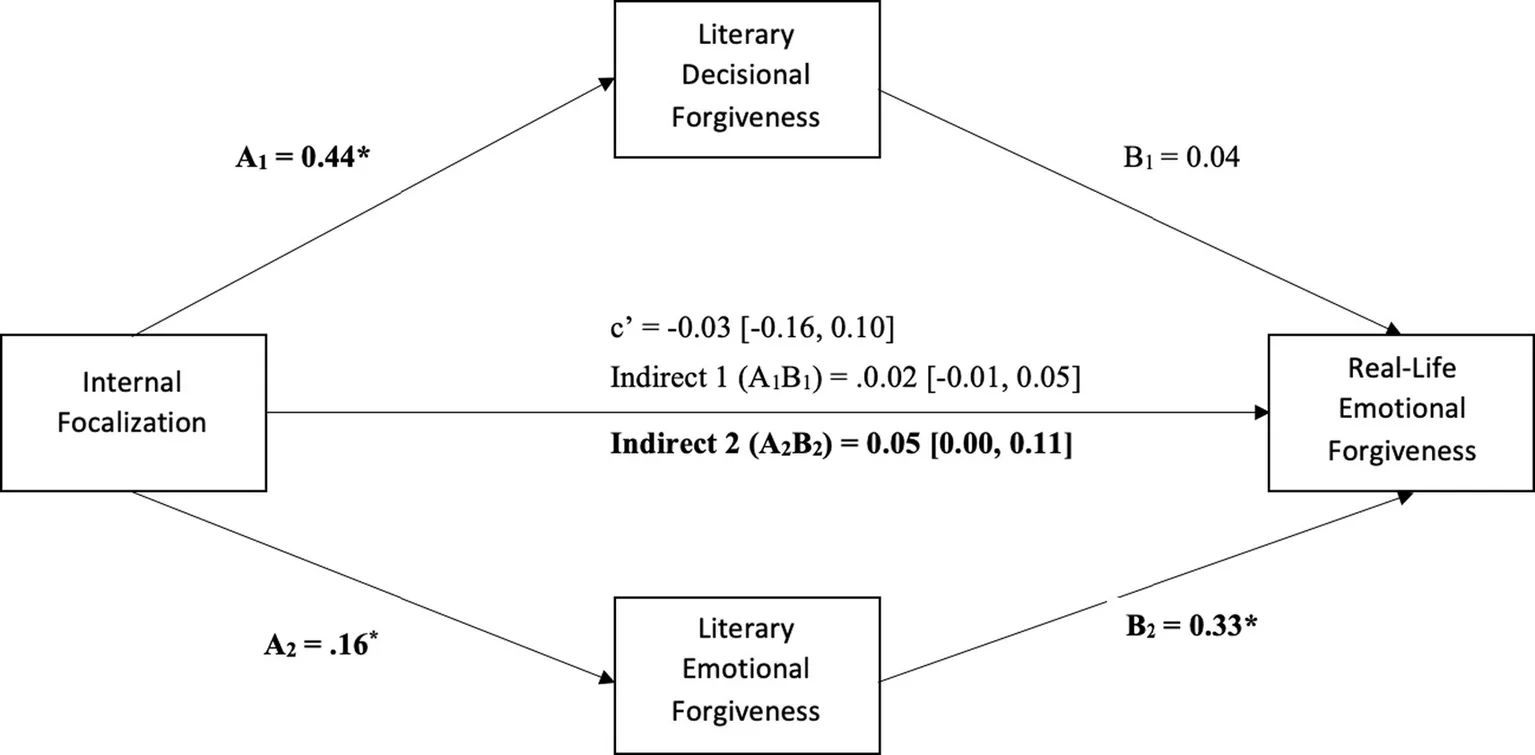
Study 2 mediation model predicting emotional forgiveness (*n* = 357). *p < 0.05.

Internal focalization improved both decisional and emotional forgiveness, replicating Study 1 with a much larger sample size, and demonstrating that internal focalization improves people’s ability to forgive in literary fiction. Moreover, internal focalization indirectly improved forgiveness towards a real-life past transgressor via increased forgiveness in the literary scenario. This finding suggests that influencing forgiveness via literature can influence the ability to forgive more broadly.

## Study 3

The purpose of Study 3 was (a) to replicate the internal focalization findings from Study 1 and Study 2 and (b) to rule out the possibility that the effects were an artifact of selecting easily forgivable past transgressions. The same method was employed as Study 2, with the addition of a hanging, no-story comparison condition, included to examine baseline real-life forgiveness (as a comparative for the story conditions). We also varied the order of presentation of the personal transgression scenario and the fictional transgression scenario, yielding a 2 (Focalization: no focalization, internal focalization) x 2 (Order: literary scenario first, personal scenario first) between-subjects design, with a hanging no-story comparison condition.

### Method

#### Participants

Amazon Mechanical Turk Participants (*N* = 961) were recruited for a study involving reading a story and answering related questions for $1.50. Participants were excluded if they did not complete the full survey, including primary dependent measures, or if they failed basic comprehension check questions related to information presented in the story (*n* = 218), yielding a total sample of *N* = 743 for analysis. The primary hypotheses centered on participants who read the literary fiction (*n* = 220 were in the hanging, no-story comparison condition), yielding 523 participants for our primary analyses.

#### Procedure

The procedure of Study 3 was the same as Study 2 with two exceptions. First, Study 3 added a hanging, no-story comparison condition to examine whether simply viewing a story improved forgiveness of past personal transgressors. Second, Study 3 varied the order of presentation for introducing the personal transgression reflection, with half of the participants asked to write about a past personal transgression prior to reading the literary fiction.

##### Hanging story control condition

Participants in the no-story comparison condition (*n* = 220) read a technical report about the fictitious company *Block Life*. The report had two parts of similar length to the first two parts of the story about Mary. To ensure that the standard of attention in the no-story condition was comparable to the story condition, participants were removed from analyses if they failed to answer basic comprehension check questions.

##### Presentation order

Participants randomly assigned to the alternative presentation order of Study 2 filled out the first half of the reflection items related to the personal transgression (including the initials of the transgressor, self-reported closeness to the transgressor, and a description of what happened and what the transgressor was thinking) prior to the literary fiction. They filled out the items related to forgiving the transgressor following the literary fiction.

#### Transparency and openness

This study’s design, hypotheses, and analyses were preregistered prior to data collection.^3^ The Institutional Review Board of The Ohio State University granted its approval for the experiment prior to the commencement of data collection and the initiation of the experimental procedures.

### Results and discussion

#### Manipulation checks

##### Mary liking

On average, participants reported positive attitudes towards Mary (*M =* 8.07, *SD* = 1.13), *t*(522) = 62.21, *Cohen’s d* = 2.72, 95% CI [2.97, 3.17]. A one-way ANOVA was computed to examine if the order of the story affected attitudes towards Mary. There was not a significant effect of order on liking Mary; participants liked Mary both when the literary fiction came first (*M* = 8.13, *SD* = 1.05) and when the personal transgression scenario came first (*M* = 8.00, *SD* = 1.21), *F* (1, 521) = 1.66, *p* = 0.198, 95% CI [−0.07, 0.32].

##### Internal focalization

Among participants who viewed the literary fiction, a two-way ANOVA was computed to examine the effects of internal focalization, order of the scenarios, and the interaction on participants’ feelings of focus on the repairman’s mind. There was a marginal main effect of story condition: Participants in the literary condition reported greater feelings of access to the repairman’s mind (*M* = 6.16, *SD* = 2.31) compared to participants in the nonliterary condition (*M* = 5.78, SD = 2.37), *F*(1,479) = 3.16, *p* = 0.076, 95% CI [−0.04, 0.80], suggesting that the focalization manipulation successfully produced internal focalization, although the effect was not as robust as in Studies 1 and 2. In sum, the literary fiction created sufficient liking for the victim while also successfully manipulating internal focalization, and this was not affected by the order of presenting the literary fiction.

#### Literary forgiveness (replicating studies 1 & 2)

For each of the primary dependent measures, a separate two-way ANOVA was used to examine the extent to which the focalization condition, order of the literary and personal scenarios, and their interaction affected both decisional and emotional forgiveness. A summary of results can be found in Table 1.

**Table 1:** Two-way ANOVAs examining effects of internal focalization and order on forgiveness (Study 3).

Factor	Decisional forgiveness			Emotional forgiveness		
	*F*(1,511)	*p*	η_p_^2^	*F*(1,471)	*p*	η_p_^2^
Literary fiction	6.19	0.013	0.012	4.41	0.036	0.009
Order	0.26	0.612	0.001	0.41	0.522	0.001
Literary fiction * order	0.48	0.490	0.001	0.11	0.739	0.000

##### Decisional forgiveness

There was a main effect of focalization on decisional forgiveness, replicating Studies 1 and 2, *F*(1,511) = 6.19, *p* = 0.013, η_p_^2^ = 0.012, 95% CI [0.13, 1.09]. Participants were more forgiving in the literary condition (*M* = 6.92, *SD* = 2.59) compared to the nonliterary condition (*M* = 6.30, *SD* = 2.93, see Figure 5).

**Figure 5:**
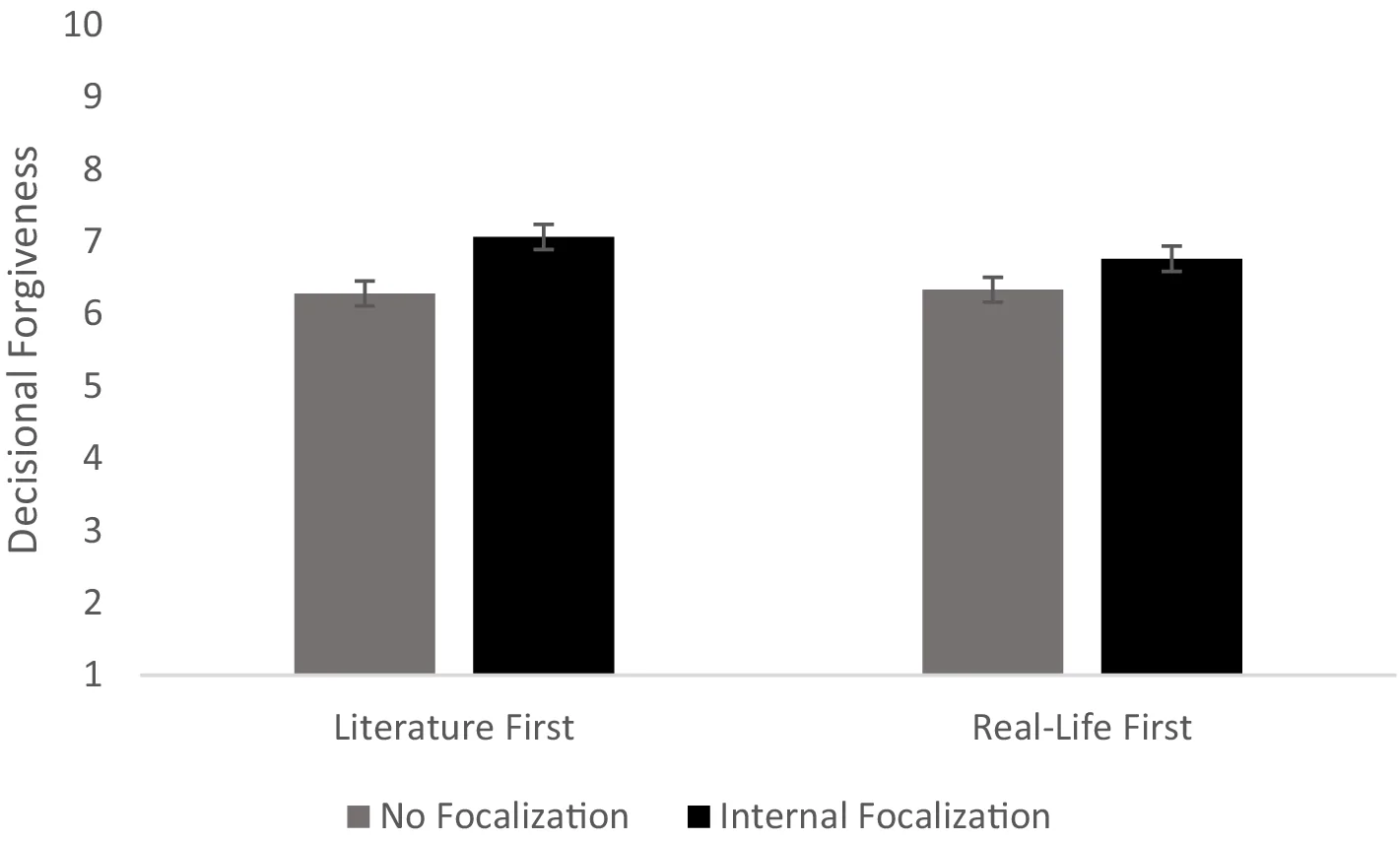
Two-way ANOVA examining the effects of order and internal focalization on decisional forgiveness (Study 3).

##### Emotional forgiveness

There was a main effect of focalization on emotional forgiveness, replicating Studies 1 and 2, *F*(1,471) = 4.41, *p* = 0.036, η_p_^2^ = 0.009, 95% CI [0.02, 0.46]. Participants were more forgiving in the literary condition (*M* = 3.15, *SD* = 1.21) compared to the nonliterary condition (*M* = 2.91, *SD* = 1.24, see Figure 6).

**Figure 6:**
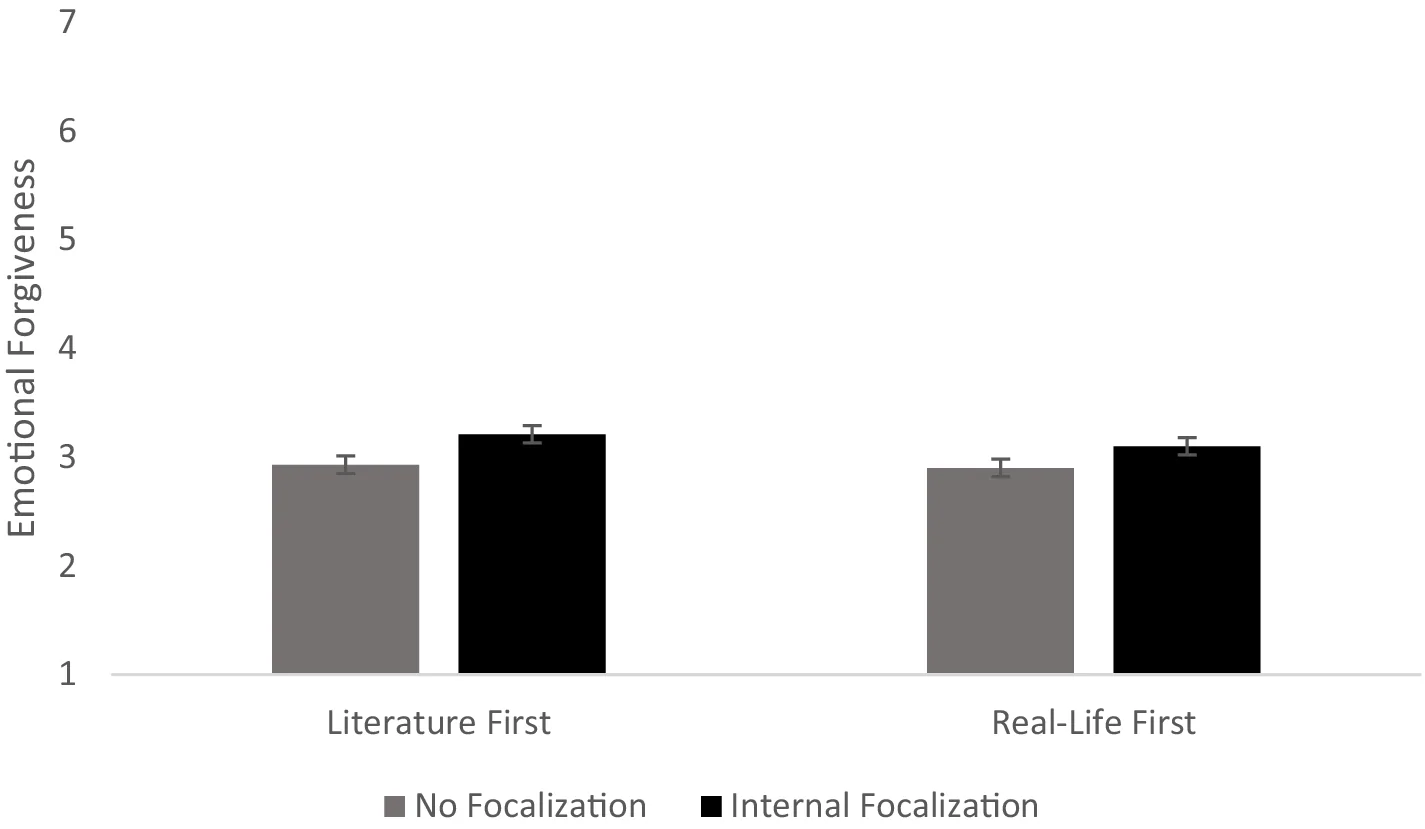
Two-way ANOVA examining the effects of order and focalization on emotional forgiveness (Study 3).

#### Real-life transgression forgiveness

We first examined whether simply reading a story compared to a technical report affected decisional and emotional forgiveness by computing One-Way ANOVAs. This possibility was excluded. We then computed the same mediation models as in Study 2 (Process V4.1, Model 4). Because the order of the stories did not have any significant effects, we did not examine order as a moderator in these analyses.

##### Real-life decisional forgiveness

Literary decisional forgiveness positively predicted real-life decisional forgiveness, *B* = 0.41, *SE* = 0.06, *t*(448) = 6.58, *p* < 0.001, 95% CI [0.29, 0.54], whereas literary emotional forgiveness did not predict real-life decisional forgiveness, *B* = 0.07, *SE* = 0.14, *t*(448) = 0.51, *p* = 0.607, 95% CI [−0.21, 0.35]. As predicted, internal focalization had a positive indirect effect on real-life decisional forgiveness via literary decisional forgiveness, *B* = 0.27, *SE* = 0.12, *t* (448) = 2.32, *p* = 0.021, *95*% CI [0.06, 0.51], replicating Study 2 (see Figure 7).

**Figure 7:**
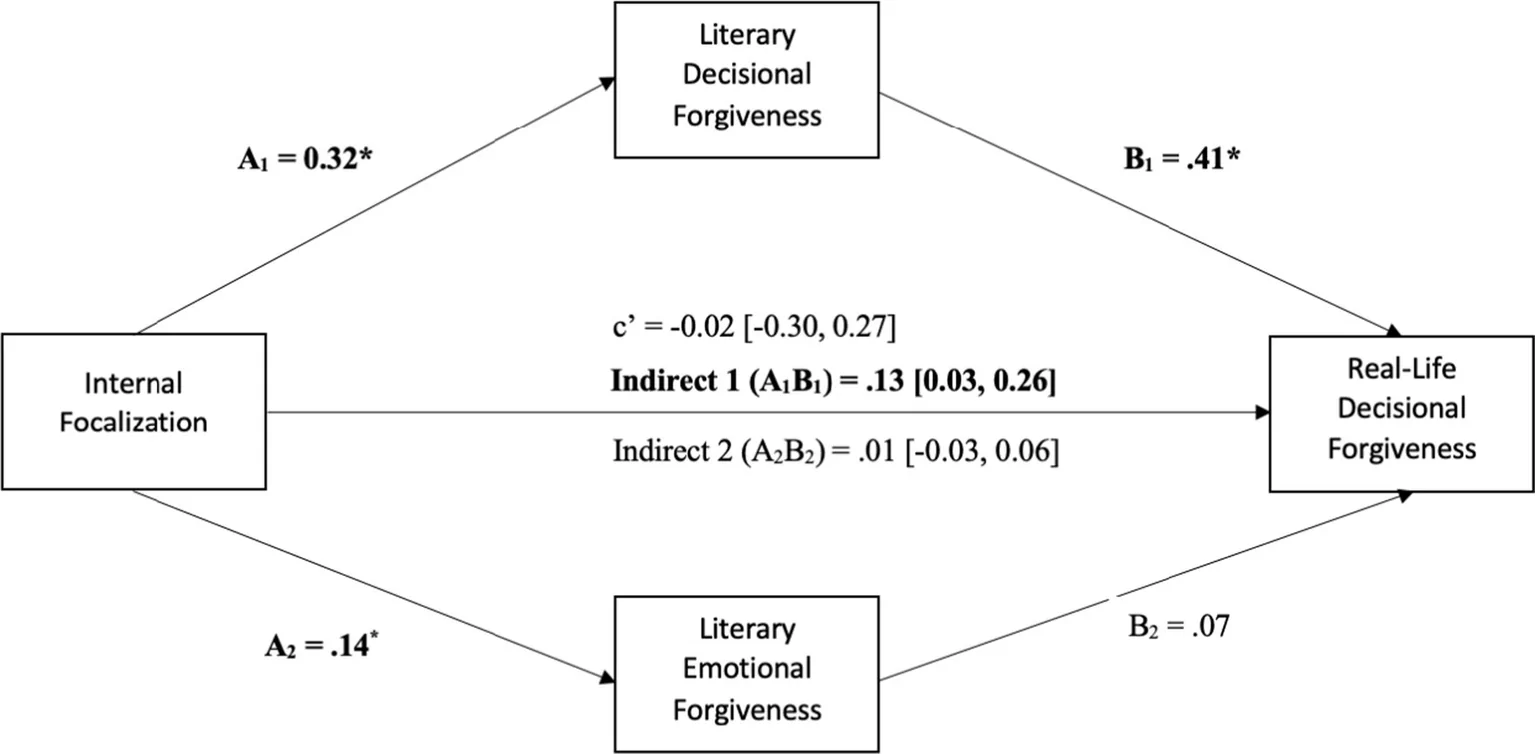
Study 3 mediation model predicting decisional forgiveness (*n* = 452). *p < 0.05.

##### Real-life emotional forgiveness

Literary emotional forgiveness positively predicted real-life emotional forgiveness, *B* = 0.29, *SE* = 0.06, *t*(421) = 4.80, *p* < 0.001, 95% CI [0.17, 0.40]. Literary decisional forgiveness also positively predicted real-life emotional forgiveness, *B* = 0.06, *SE* = 0.03, *t*(421) = 2.19, *p* = 0.029, 95% CI [0.01, 0.11]. As predicted, internal focalization had a positive indirect effect on real-life emotional forgiveness via literary emotional forgiveness, *B* = 0.09, *SE* = 0.04, *t*(421) = 2.13, *p* = 0.034, 95% CI [0.03, 0.22], replicating Study 2 (see Figure 8).

**Figure 8:**
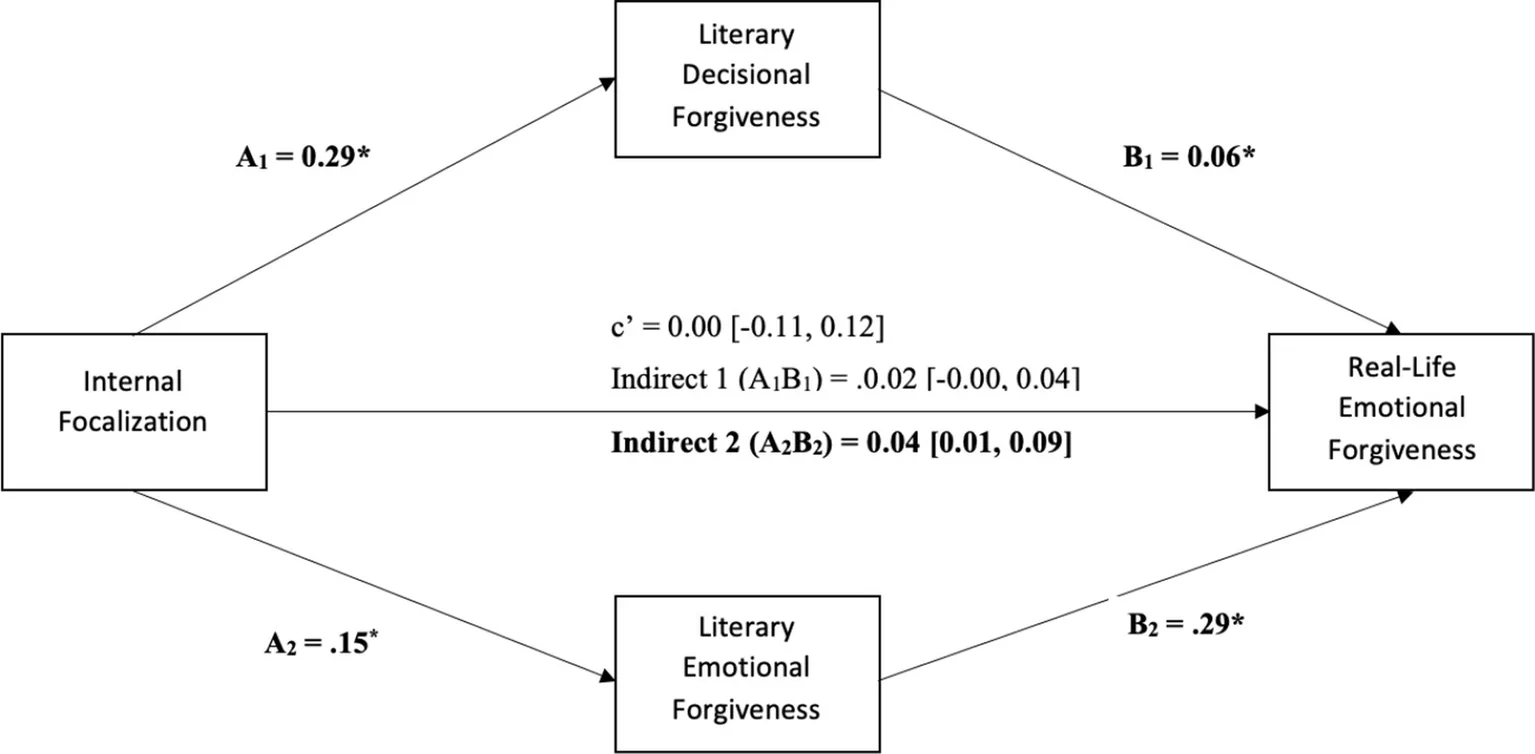
Study 3 mediation model predicting emotional forgiveness (*n* = 425). *p < 0.05.

The results of Studies 1 and 2 were fully replicated in Study 3. Participants were more forgiving in the literary scenario. Internal focalization indirectly facilitated forgiveness of a past personal transgression by improving the ability to forgive in literary fiction.

## General discussion

Forgiveness is a socio-emotional process that promotes healthy relationships and personal wellbeing. Across three studies, we found that forgiveness can be promoted via the literary technique of internal focalization. Moreover, in Studies 2 and 3, we found that facilitating literary forgiveness can facilitate forgiveness of real-life transgressions.

To our knowledge, this is the first study to demonstrate empirically how forgiveness can be facilitated by fiction. In fiction, forgiveness can be facilitated, as in real life, by conveying information that affords external attributions towards the behavior (Study 1; Takaku, 2001). But forgiveness can also be facilitated via a literary technique, internal focalization, that increases trust in the transgressor’s remorse (Studies 1–3).

This is also the first study to demonstrate how forgiveness experienced within a literary scenario can promote forgiveness of past transgressors. This effect is both novel and notable: Brief exposure to literature presented in an online experiment led to changes in real-life forgiveness. This effect occurred while readers reflected on a *severe* past personal transgression, where forgiveness is difficult (Fincham et al., 2005; Fernández-Miranda et al., 2026).

Finally, in Studies 2 and 3, forgiveness of a literary character promoted both decisional and emotional forgiveness of real-life transgressors, meeting two conditions necessary for repairing safety and connection in a relationship (Worthington et al., 2007). This provides evidence for the mental health utility of a literature-based forgiveness intervention.

## Future directions

The present research identifies a literary technique—internal focalization—that influences the emotional processing of difficult past events, potentially improving mental and social wellbeing. Future research could expand on the mechanisms underlying literary forgiveness, assess the health benefits of literary forgiveness, and explore the role of literary fiction in helping people forgive in ways that do not risk further harm (i.e., *How do I forgive without needing to continue the relationship?* Krause and Rucker, 2020). Future research could also explore other aspects of empathy or could move beyond empathy to explore courage, love, grief-processing, and other mental health benefits that have been traced to distinct literary inventions (Fletcher, 2021).

## Constraints on generality

All our samples were taken from online sources that tend to reflect Western populations. It is possible, and likely, that different groups or cultures will have customs that either facilitate or inhibit forgiveness relative to our samples. For example, some traditional Hawaiian cultures have a tradition of proactive forgiveness (i.e., *Ho’oponopono*). Perhaps in such a cultural setting, forgiveness may be easier to facilitate (Ito, 1985). On the other hand, people from groups centered on a “Culture of Honor” that encourages members to defend their pride (Ceylan-Batur et al., 2023) might show greater resistance to forgiveness. Moreover, some cultures may be more or less likely to see fictional experience as applicable to real life, which could influence the likelihood of fictional experiences impacting real-life processing.

## Conclusion

Literature is not reducible to a single aesthetic form. It is a library of many formal techniques, each rooted in a creative response to a distinct anthropological challenge. Not all these techniques will yield the same (or any) health benefits. But by indicating that one such technique can influence real-life empathy, the present research suggests the value of isolating individual formal inventions, allowing for more precise evaluation of literature’s potential health benefits.

## Data Availability

The raw data supporting the conclusions of this article will be made available by the authors, without undue reservation.

## Footnotes

1
https://osf.io/35wq6/?view_only=c4988b8172fa46239b5f5edb322d7f7f

2
https://osf.io/tsaqb/?view_only=4f13eac0fdc740cf9942aa7b9a3627f1

3
https://osf.io/jt5gb/?view_only=b2364710f676452eab5be3ca8dc3103a
